# Neutrophil Extracellular Traps Promote T Cell Exhaustion in the Tumor Microenvironment

**DOI:** 10.3389/fimmu.2021.785222

**Published:** 2021-11-24

**Authors:** Christof Kaltenmeier, Hamza O. Yazdani, Kristin Morder, David A. Geller, Richard L. Simmons, Samer Tohme

**Affiliations:** Department of Surgery, University of Pittsburgh, Pittsburgh, PA, United States

**Keywords:** neutrophil extracellular traps, tumor microenvironment, T cell exhaustion, T cell dysfunction, program death-ligand 1, PD-L1

## Abstract

While neutrophil extracellular traps (NETs) are important for directly promoting cancer growth, little is known about their impact on immune cells within the tumor microenvironment (TME). We hypothesize that NETs can directly interact with infiltrating T cells to promote an immunosuppressive TME. Herein, to induce a NET-rich TME, we performed liver Ischemia/Reperfusion (I/R) in an established cancer metastasis model or directly injected NETs in subcutaneous tumors. In this NET-rich TME, the majority of CD4+ and CD8+ tumor infiltrating lymphocytes expressed multiple inhibitory receptors, in addition these cells showed a functional and metabolic exhausted phenotype. Targeting of NETs *in vivo* by treating mice with DNAse lead to decreased tumor growth, decreased NET formation and higher levels of functioning T cells. *In vitro*, NETs contained the immunosuppressive ligand PD-L1 responsible for T cell exhaustion and dysfunction; an effect abrogated by using PD-L1 KO NETs or culturing NETs with PD-1 KO T cells. Furthermore, we found elevated levels of sPDL-1 and MPO-DNA, a NET marker, in the serum of patients undergoing surgery for colorectal liver metastases resection. Neutrophils isolated from patients after surgery were primed to form NETs and induced exhaustion and dysfunction of human CD4^+^ and CD8^+^ T cells. We next targeted PD-L1 *in vivo* by injecting a blocking antibody during liver I/R. A single dose of anti-PD-L1 during surgery lead to diminished tumors at 3 weeks and functional T cells in the TME. Our data thus reveal that NETs have the capability of suppressing T cell responses through metabolic and functional exhaustion and thereby promote tumor growth. Furthermore, targeting of PD-L1 containing NETs at time of surgery with DNAse or anti-PD-L1 lead to diminished tumor growth, which represents a novel and viable strategy for sustaining immune competence within the TME.

## Introduction

Solid tumors accumulate a complex array of inflammatory cells of both innate and acquired immunity within the tumor microenvironment (TME). It is generally agreed that tumors heavily infiltrated with neutrophils carry a relatively poor prognosis (1). Cancer cells under the conditions of hypoxia or when exposed to the effects of surgery, radiation or chemotherapy produce chemokines which drive the infiltration of neutrophils, which themselves secrete chemokines contributing to additional feed forward inflammatory properties (2). Especially important, is the ability of neutrophils to form neutrophil extracellular traps (NETs), by which the nuclear DNA of the neutrophil becomes citrullinated, enlarges and ruptures the cell membrane (3). The nucleus then bursts into the extracellular space in the form of chromatin tangles dragging along an array of proinflammatory cytoplasmic and granular contents, some of which become embedded into the chromatin.

NETs were originally thought to serve a microbicidal function by enmeshing invading microbes within the chromatin, but it is now clear that NETs can elicit and propagate sterile inflammation and thrombosis leading to bystander local host tissue damage and even activate a number of systemic pathophysiological functions (4–6). Many studies have shown that NETs possess numerous powerful pro-tumorigenic properties (7, 8). They are instrumental in the establishment of pre-metastatic niches, the awakening of dormant metastases and can directly enhance cancer growth (7, 9–12). NETs can promote capture, invasion, and migration of circulating tumor cells. In addition, NETs have also been shown to directly promote mitochondrial biogenesis and proliferation of tumor cells *in vitro* even in the absence of other inflammatory cells (13).

Comparative to the direct effect of NETs on cancer cells, much less is known about the interaction of NETs and the infiltrating immune cells in the TME. Recently, within the TME, NETs have been shown to physically shield tumor cells from cytotoxic T and NK cells by surrounding the malignant tumor cells (14). Among the cells infiltrating the TME, T cells are most commonly affected as they can be rendered non-functional with chronic antigen stimulation leaving them in a state of exhaustion (15). These exhausted T cells show overexpression of inhibitory receptors, decreased effector cytokine production and cytolytic activity, leading to the failure of cancer elimination. The tumor mediated pathways of T cell exhaustion have been well studied, however the interplay and exact mechanism of the crosstalk between the adaptive and innate immune system in the TME leading to T cell dysfunction remains unknown. Understanding the exact mechanism of T cell exhaustion, mediated by innate immune cells in the TME with an overarching goal to restore T cell function, represents an inspiring strategy for cancer treatment. We hypothesized that NETs may play an important role in inducing exhaustion and dysfunction of T cells in the TME, permitting immune escape and augmented tumor growth. Here, to address this crosstalk, we directly examined the phenotype, metabolism and functional capacity of tumor infiltrating T cells in the presence of NETs using murine tumor models and *in vitro* studies. Understanding the direct cell-cell interactions between the innate and adaptive immunity leading to exhaustion have crucial implications for the success of checkpoint blockade and other strategies to increase the effector function of T cells and potential clinical implications for the treatment of cancer patients.

## Methods

### Cell Lines

MC38 (murine colorectal adenocarcinoma) cell line were obtained from Dr. Michael Lotze (University of Pittsburgh, Pittsburgh, PA). Cell lines were amplified in our laboratory and stored in liquid nitrogen to ensure that cells used for experiments were passaged for fewer than 6 months. Cell lines were tested biannually for identity by appearance and growth curve analysis and validated to be mycoplasma free.

### Animals

Male 8–12-week-old wildtype (C57BL/6) and PD-1 KO mice (B6.Cg-*Pdcd1^tm1.1Shr^*/J) were purchased from Jackson Laboratories. PD-L1 knockout (PD-L1 -/-) mice were kindly provided from Dr. Dong at Mayo Clinic, Rochester, Minnesota. All mice were maintained under pathogen-free conditions and the experiments were performed according to protocols approved by Institutional Animal Care and Use Committee of the University of Pittsburgh.

### Tumor Models and Liver Ischemia/Reperfusion Injury

For the colorectal liver metastases model, 1x10^6^ MC38 cells were injected *via* the portal vein using a 31g needle (TSK). The micrometastases were allowed to grow in the liver for 5 days prior to performing liver ischemia/reperfusion (IR). On day 6, 70% non-lethal hepatic ischemia and reperfusion was performed as described previously ^16^. Briefly, the median, lateral and quadrate lobes of the liver were deprived of blood flow by occluding the portal triad to those segments by placing a vascular clamp. Reperfusion was induced after 60 mins by removing the clamp. Intraperitoneal administration of DNAse (Sigma-Aldrich) or anti-PD-L1 (Bioxcell, clone: 10F.9G2) was performed prior to inducing ischemia and at daily intervals after the liver was reperfused. Bilateral subcutaneous tumors were established after injection of 1x10^6^ MC38 cells in each flank of healthy mice. This was followed by bi-weekly injections of either NETs or PBS into the palpable tumor.

### Tissue Harvest

Cardiac puncture was used to collect blood prior to sacrificing animals. Organs were flushed with PBS, harvested and further prepared for subsequent studies. Single cells suspensions of harvested organs were obtained by digestion with 1mg/ml collagenase IV (Sigma-Aldrich)) and 2U/L dispase (Sigma-Aldrich). Cells were filtered through a 70uM cell strainer and a Ficoll (Thermo Fisher Scientific) gradient was used to isolate leukocytes.

### Immunofluorescent Imaging

Liver and subcutaneous tumors were harvested from the mice. Sample sections were incubated with antibodies against PD-L1 (clone MIH5, Novus Biologicals), citH-3 (ab5103, Abcam) and CD66b (Thermo Fisher Scientific). Coverslip plated cells were treated similarly to *in vivo* samples. Secondary antibodies were added for 1h at room temperature. Cell nuclei were stained with 1 mg/ml Hoechst (Center for Biologic Imaging, Pittsburgh) or DAPI (Sigma-Aldrich). Imaging was performed using an Olympus Fluoview 1000 microscope.

### Neutrophil Isolation, NET Induction and Quantification

Murine neutrophils were isolated following harvest of bone marrow from 8-week-old naïve B6 or PD-L1 KO mice. In brief, skin, muscle and fat were removed from femurs and tibias. Bone marrow was then flushed using a 27g needle and RPMI (Gibco). Following isolation of pure neutrophils, cells were then treated with 250-500 nM phorbol-12-myristate-13-acetale (PMA, Sigma-Aldrich) for 4 hours and then spun at 480xg for 5 minutes. The pellet was discarded and supernatant containing NET chromatin was spun at 18,000xg for 10 minutes. Quantification of NETs was analyzed using a Nano Drop (Thermo Fisher Scientific). *In-vivo* NETs quantification was analyzed using myeloperoxidase (MPO) (Roche) associated with DNA ELISA (Roche). For the human studies, fresh peripheral blood obtained from healthy donors or patients was stained with anti-CD15 (clone W6D3, BioLegend), anti-CD16 (clone 3G8, BioLegend), anti-citrullinated histone H3 (Abcam), anti-PD-L1 (clone 29E.2A3, Biolegend) and specific secondary anti-antibodies (Thermo Fisher Scientific).

### *In Vitro* T Cell Stimulation

Splenic CD4^+^ or CD8^+^ T cells were isolated from 8-week-old B6 mice or PD-1 KO mice. Tissue was harvested, mechanically disrupted and filtered through a 70uM cell strainer. Red blood cells were lysed using Red Blood Cell lysis buffer (Sigma). Cells were stained with surface markers for CD3 (clone 17A2, BioLegend), CD4 (clone GK1.5, BioLegend) and CD8a (clone 53-6.7, BioLegend) for sorting using a BD FACSAria III (BDBiosciences). 100,000 cells were seeded in 96 round u-wells and treated with CD3/28 Dynabeads Mouse T-activator (Thermo Fisher Scientific) in the presence or absence of WT NETs or PD-L1 KO NETs.

### T Cell Assays

*Proliferation:* Single-cell suspensions made from spleen or tumor were loaded with 5 μM Cell Trace Proliferation Dye (Thermo Fisher Scientific) according to the manufacturer’s instructions (20 minutes at RT in PBS). They were then quenched on ice with AIM V (Gibco, Thermo Fisher Scientific) supplemented with 5% FCS (Thermo Fisher Scientific), penicillin and streptomycin (Gibco, Thermo Fisher Scientific Scientific), 10 mM HEPES (Gibco, Thermo Fisher Scientific Scientific), and 2 mM l-glutamine (Gibco, Thermo Fisher Scientific Scientific) for 5 minutes. Cells were then treated with CD3/28 Dynabeads in the presence of IL-2 (100 IU/ml) for indicated times. Cells were then analyzed using flow cytometry. *In vitro* proliferation was also determined by WST-8 (2-(2-methoxy-4-nitrophenyl) - 3 - (4-nitrophenyl) - 5 -(2, 4-disulfophenyl) - 2 H - tetrazolium, monosodium salt), assay using a Cell Counting Kit -8 (CCK-8, Dojindo Laboratories) according to the manufacturers’ instructions.

*Intracellular cytokines:* For intracellular cytokine staining, single-cell suspensions of cells were stimulated *in vitro* with 50 ng/ml PMA (Sigma Aldrich) and 1 μg/ml ionomycin (Sigma Aldrich) for 4 hours in the presence of 1:1000 Brefeldin A (BioLegend. Intracellular staining was performed using Fixation/Permeabilization kit (BD Bioscience). Permeabilized cells were then stained for IL-2 (JES6-5H4, BioLegend), TNF-α (MP6-XT22, BioLegend) and IFN-γ (D8-1, BioLegend and analyzed by flow cytometry as described above.

*Metabolism:* Single-cell suspension from tissue or sorted CD4^+^ or CD8^+^ T cells were stained with MitoTracker Deep Red FM (Thermo Fisher Scientific), tetramethylrhodamine ester (Thermo Fisher Scientific), 2-NBDG (Thermo Fisher Scientific) or Bodipy (Thermo Fisher Scientific). In brief cells were pulsed with 50-100 μM NBDG for 30 min at 37°C prior to staining with 50 nM Mitotracker, 2.5 μM Bodipy or 50 nM TMRE.

### Patient Samples and Data

Patients with metastatic colorectal carcinoma were studied prior to partial hepatectomy designed to remove all known liver metastatic disease. All human materials utilized during these studied were obtained under the University of Pittsburgh institutional review board (IRB) [Protocol# MOD08010372-28/PRO08010372 after written informed consent. Serum and whole blood samples were obtained pre-operatively on the day of surgery. Post-operative samples were obtained on day 1 following partial hepatectomy.

### Statistical Analysis

For animal studies results are expressed as standard error of the mean (SEM). Paired 2-tailed Student’s *t* test or ANOVA analysis was performed where indicated. Each experiment was replicated at least 2 times. Shown is representative of at least 2 independent experiments unless otherwise indicated. *P* < 0.05 was considered statistically significant, although lower *P* values are indicated in individual figure legends.

## Results

### T Cells in a NET-Rich Murine Tumor Environment Exhibit A Phenotype of T Cell Exhaustion and Dysfunction

**Figure 1:**
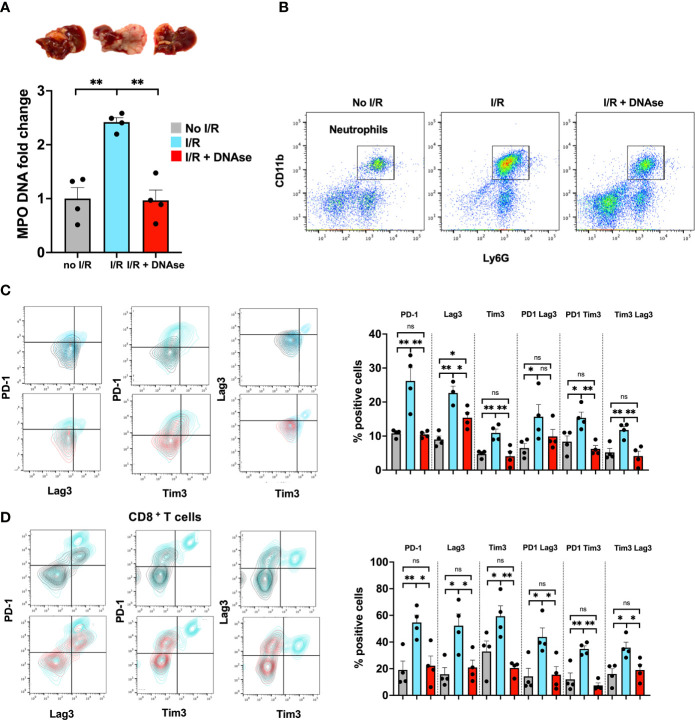
T cells in a NET-rich environment exhibit a phenotype of T cell exhaustion. Mice underwent tumor inoculation followed by liver I/R on day 6 or sham treatment. Animals treated with DNAse received daily injection until tumor harvest 3 weeks later. **(A)** Animals that underwent I/R had elevated serum MPO-DNA levels and massive tumors. Sham mice (no I/R) and I/R mice treated with DNAse had normal levels of MPO-DNA and smaller tumors. **(B)** Similarly, higher levels of Neutrophils were found in the TME of I/R animals. **(C, D)** T cells within the TME of mice that underwent I/R express higher levels of markers for T cell exhaustion including PD1, LAG3, Tim3 and double positive markers compared to sham animals. T cell exhaustion is reversed to baseline in animals that underwent I/R and were treated with DNAse. Representative flow plots for double stained cells are shown. Bar graphs represent the mean +/- SEM of three individual experiments performed in duplicates or triplicates; *p < 0.05/**p < 0.01).

Numerous studies have shown that tumor progression is positively correlated with a TME rich in neutrophils and neutrophil extracellular traps; however less is known about the crosstalk between NETs and T cells (16). Five days following tumor inoculation *via* the portal vein, we performed liver I/R which promotes neutrophil infiltration to the liver and formation of NETs (measured by quantitating serum MPO, tissue cit-H3 and flow cytometry). By 3 weeks massive metastatic tumor growth was found. All these changes were minimized in mice treated with DNAse, which is known to digest NETs and inhibit the function of NET-embedded proteins (**Figures 1A, B**).

**Figure 2:**
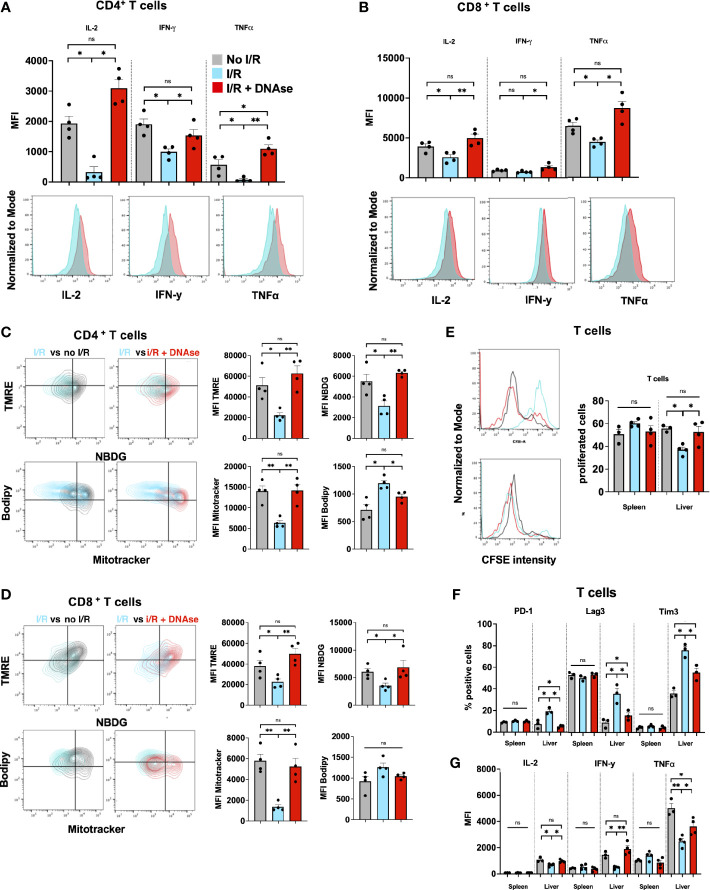
T cells in a NET-rich environment exhibit functional exhaustion. **(A, B)** Following I/R, T cells infiltrating the TME express reduced T cell cytokine levels; the changes return to sham levels after treatment with DNAse. Representative flow cytometry plots comparing CD4^+^ **(A)** or CD8^+^ **(B)** T cells in IR vs IR + DNAse are shown. **(C, D)** Following I/R, T cells in the TME have altered metabolic function measured by TMRE, Mitotracker, Bodipy and NBDG. These metabolic effects on T cell metabolism are reversed in animals treated with I/R + DNAse; Representative flow plots are shown comparing No I/R vs I/R and I/R vs I/R + DNAse. **(E)** T cells from mice that underwent No I/R, I/R or I/R + DNAse treatment were cultured *in vitro*. Proliferation was assessed following staining with a CFSE proliferation dye and 5 days of stimulation with anti-CD3/anti-CD28 beads. **(F, G)** Following I/R T cells infiltrating the TME express higher levels of exhaustion markers and lower levels of cytokines, these effects are reversed with DNAse. T cells in the spleen are unaffected by liver I/R or DNAse administration. (bar graphs represent the mean +/- SEM of three individual experiments performed in duplicates or triplicates: *p < 0.05/**p < 0.01). ns, not significant.

We hypothesized that tumor infiltrating lymphocytes of these large metastatic tumors containing NET-rich infiltrates would exhibit an exhausted phenotype. T cells, both CD4^+^ and CD8^+^, in the NET-rich TME of animals that underwent I/R expressed significantly higher levels of markers of exhaustion (PD-1, Tim3, Lag3) compared to the tumor infiltrating T cells in mice who had not been subjected to I/R or who had received daily DNAse injection in addition to I/R (**Figures 1C, D**). These isolated T cells also exhibited diminished cytokine production and a profoundly altered metabolic profile with decreased mitochondrial function and mass as well as decreased glucose uptake and increased fatty acid uptake (**Figures 2A–D**). In addition, *in vitro*, a decreased proliferative capacity in response to stimulation with CD3/28 beads was observed (**Figure 2E**). All of these factors, indicative of T cell exhaustion and dysfunction in the NET-rich TME of large tumors in mice, were reversed by DNAse treatment. Evidence of exhaustion was only observed among the T cells infiltrating the TME together with neutrophils and NETs following liver I/R. In contrast, splenic T cells did not express exhaustion markers (**Figure 2F**) and maintained normal function in all treated animals and conditions (**Figure 2G**). These findings were consistent with the idea that a NET-rich environment is responsible for loss of T cell function in the TME.

### Exogenous Administration of NETs Leads to Enhanced T Cell Exhaustion in the TME

**Figure 3:**
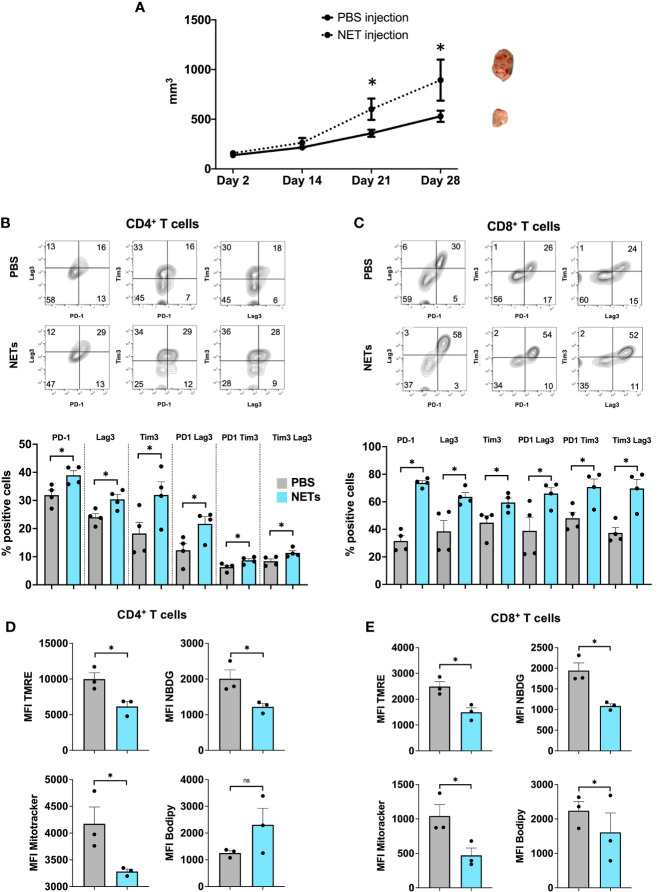
Exogenous administration of NETs leads to enhanced T cell exhaustion in the TME. **(A)** 8-week-old B6 mice underwent subcutaneous injection of MC38 cancer cells in addition to biweekly NETs or PBS, respectively. Mice following NET injection had larger tumors at 3-4 weeks. **(B, C)** Markers for T cell exhaustion were analyzed by flow cytometry, these markers were significantly upregulated on T cells in tumors that received additional injection or purified NET chromatin but not PBS. **(D, E)** Similarly, T cells in NET injected tumors showed decreased mitochondrial activity, decreased glucose but increased lipid uptake compared to PBS injected tumors. Bar graphs represent mean +/- SEM of two independent experiments, performed with 3 mice each. *p < 0.05, **p < 0.01.

The prior used *in vivo* model demonstrated that tumor infiltrating lymphocytes in the NET-rich lobes expressed a phenotype of T cell exhaustion. This model yields a complex immunothrombotic response with hepatic bystander damage, we therefore adopted a second tumor model with injection of NETs or PBS into subcutaneous tumors. Here, we found that mice injected with NETs, but not PBS, had markedly increased tumor growth by 3 weeks (**Figure 3A**). Both, CD4^+^ as well as CD8^+^ T cells in the TME of NET injected tumors expressed significantly higher levels of both phenotypic (**Figures 3B, C**) and metabolic exhaustion (**Figure 3E**). These results are in accord with T cell exhaustion found within NET-rich hepatic metastases (**Figures 1**, **2**).

### NETs Directly Promote T Cell Exhaustion *In Vitro*

**Figure 4:**
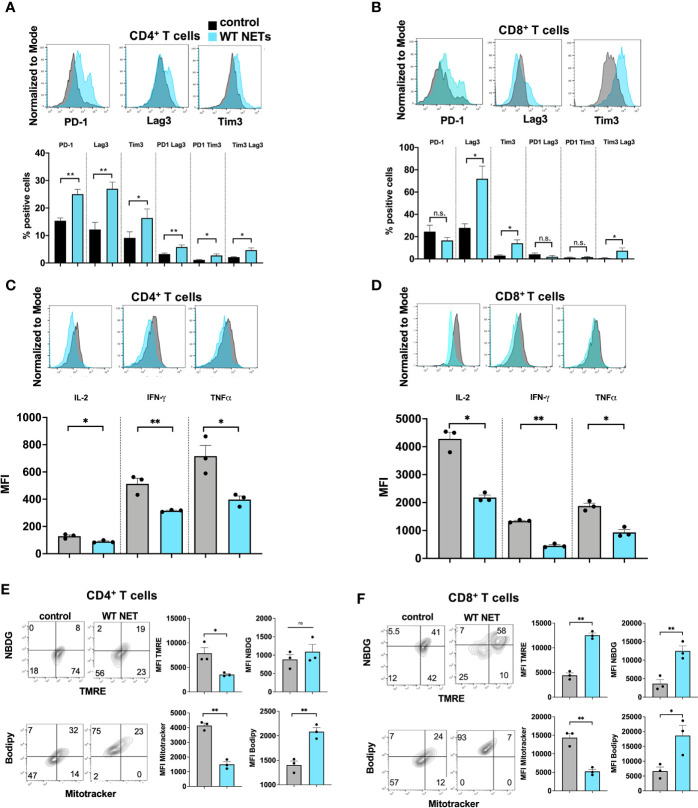
NETs cause phenotypic, metabolic and functional T cell exhaustion *in vitro.* Splenic CD4^+^ T cells were pretreated with CD3/28 beads and then cultured for 72h in the presence of isolated WT NETs. **(A, B)** Markers for T cell exhaustion analyzed by flow cytometry were significantly upregulated on T cells in the presence of WT NETs. **(C, D)** T cell intracellular cytokines were analyzed by flow cytometry following fixation and permeabilization. T cells in the presence of WT NETs expressed significantly lower levels of intracellular cytokines. **(E, F)** Similarly, following culture with WT NETs, T cells were stained with metabolic markers, showing decreased metabolic activity in the presence of WT NETs. Bar graphs represent mean +/- SEM of three independent experiments, performed in duplicates or triplicates. *p < 0.05, **p < 0.01.

Thus far, we had shown that a NET rich environment leads to accelerated metastatic tumor growth and T cells that show a phenotype of exhaustion. The effect of T cell exhaustion was abrogated with the use of DNAse to eliminate NET contents in the TME. To further assess the direct effects of NETs on T cells, we cultured normal T cells in the presence of isolated NETs from WT mice *in vitro.* Following exposure of T cells to WT NETs we found that the T cell exhaustive phenotypic changes were similar to those found *in vivo* within the NET-rich TME. T cells cocultured with WT NETs were found to upregulate surface markers of exhaustion including PD-1, Tim3 and Lag3 (**Figures 4A, B**). This effect was reversed in the presence of DNAse (**Suppl. Figure 1D**). In addition, culture of T cells with WT NETs decreased T cell cytokine expression levels to levels similar to those T cells found within the large NET-rich tumor in I/R treated mice (**Figures 4C, D**). Furthermore, T cells cultured with WT NETs were found to change their metabolic profile leading to decreased mitochondrial function (TMRE), decreased mitochondrial mass (Mitotracker), decreased glucose (NDBG) and increased lipid (Bodipy) uptake (**Figures 4E, F**).

### PD-L1 Is Contained Within NETs Derived From Murine and Human Neutrophils

**Figure 5:**
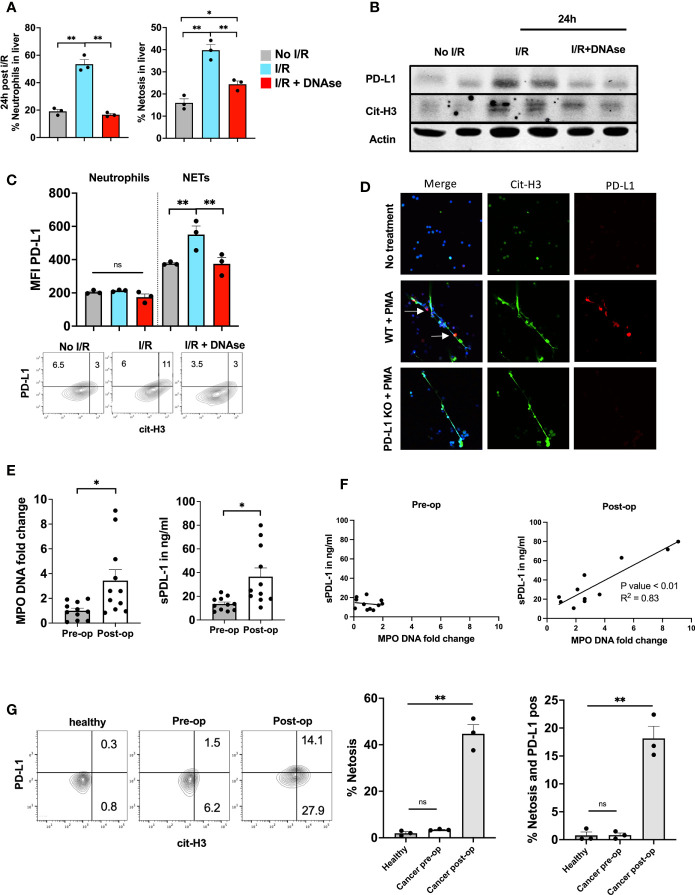
PD-L1 is embedded inside NET chromatin. **(A)** 8-week-old B6 mice underwent liver I/R without tumor injection followed by harvest of the ischemic lobe at 24h. Tissue was analyzed by flow cytometry for the presence of neutrophils and netosis. The ischemic lobe was extensively infiltrated by neutrophils and NETs unless DNAse was administered. **(B)** Western blot of ischemic lobes was performed showing increased levels of PD-L1 and cit-H3 in mice that underwent liver I/R This effect was reversed by treatment with DNAse. **(C)** Neutrophils within the ischemic lobe were stained for PD-L1 or PD-L1 and cit-H3. **(D)** Immunofluorescence staining shows that treatment of normal WT bone marrow derived neutrophils with PMA leads to the upregulation of PD-L1 within the extruded NET chromatin; however, no PD-L1 expression was found in PD-L1 KO NETs. Flow cytometry of isolated WT bone marrow derived Neutrophils stained with cit-H3 and PD-L1 are shown. **(E)** Patient serum levels of both MPO-DNA and sPD-L1 were elevated in the first post-operative days after hepatic resection. **(F)** MPO-DNA and sPD-L1 levels were correlated pre-/and postoperative. **(G)** Whole blood from healthy individuals or patients undergoing surgery was stained for the presence of neutrophil forming NETs. A greater percentage of NET forming neutrophils was found in patients on post-operative day 1 compared to pre-operative and healthy individuals. Similarly, more NETs were present and expressed PD-L1 in the post-operative patient. (n=3) Flow cytometry plots are representative of one experiment. Bar graphs represent mean +/- SEM of two independent experiments. MPO-DNA/s-PD-L1 levels **(E, F)** were derived from eleven healthy or postoperative patients. **(G)** three healthy or postoperative patients were analyzed for neutrophil NET markers and surface PD-L1 expression. *p < 0.05, **p < 0.01.

In parallel to the increased NET levels and T cell dysfunction in the TME at 3 weeks we also found increased levels of neutrophils, NETs and PD-L1 expressed within the liver 24h post I/R in non-tumor bearing mice. This effect was reversed with the administration of DNAse (**Figure 5A**). In addition, western blot protein analysis of the ischemic lobes 24h following I/R showed high paralleled expression levels of cit-H3 and PD-L1 (**Figure 5B**). Due to the high levels of NETs within the liver following I/R and the increased levels of PD-L1 that were abrogated using DNAse (**Figure 4B**), we hypothesized that PD-L1 is embedded within the NET chromatin. Flow cytometry analysis of the infiltrating neutrophils within the liver following I/R showed that the expression of PD-L1 on the cell surface of neutrophils did not differ between groups (**Figure 5C** left panel). However, upon costaining for NETs and PD-L1, we found that NETs were the main source of PD-L1 found within the liver following I/R (**Figure 5C** right panel). To confirm the presence of PD-L1 in NETs *in vitro*, we treated bone marrow derived neutrophils from WT or PD-L1 KO mice *in vitro* with PMA to promote NET formation. PD-L1 and cit-H3 were found to be elevated in parallel when NETosis occurred in WT mice. NETs from PD-L1 KO mice were used as a negative control for IF staining (**Figure 5D**).

Next, we collected pre- and post-operative blood samples from patients who underwent partial liver resection for colorectal liver metastasis to analyze levels of MPO-DNA and soluble PD-L1 (sPD-L1). Pre-operative levels of MPO-DNA and sPD-L1 were similar to those in healthy volunteers. Post-operative day 1 serum levels of MPO-DNA, a reliable marker for NETosis, were significantly elevated compared to pre-operative levels, as were sPD-L1 levels (**Figure 4E**). We furthermore found expression of PD-L1 embedded into NET chromatin on immunofluorescence staining (**Suppl. Figure 1A**). **Figure 5F** shows that there was a significant correlation between these levels in the post-operative period. Circulating neutrophils undergoing NETosis were found in the blood of post-operative patients and these NETs stained positively for PD-L1 (**Figure 5G**).

### PD-L1 Contained Within NETs Is Responsible for T Cell Exhaustion *In Vitro*

**Figure 6:**
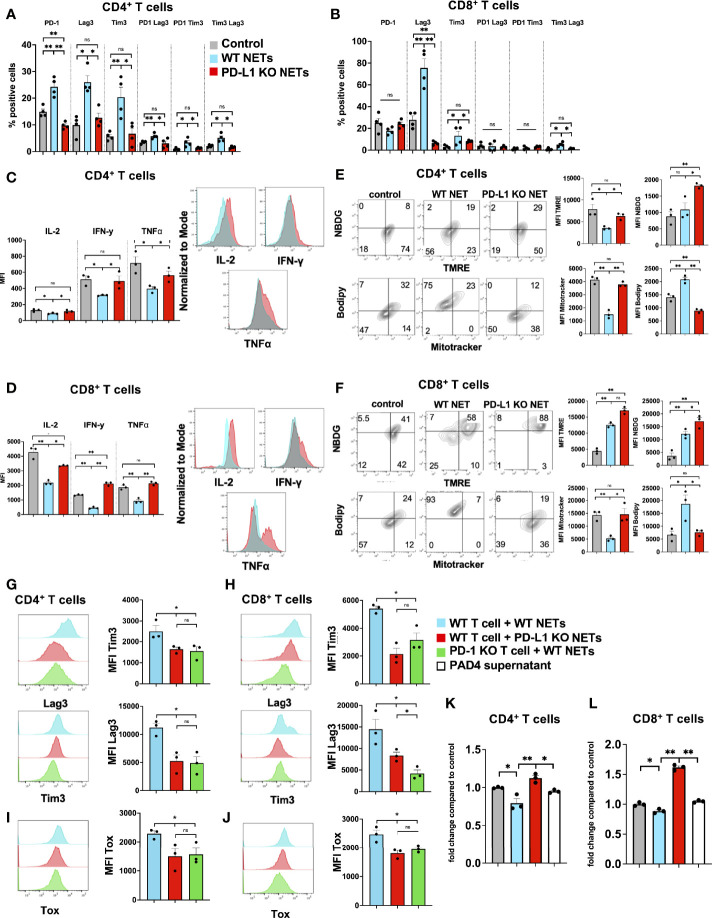
PD-L1 within NETs causes phenotypic, metabolic and functional T cell exhaustion *in vitro.* Splenic CD4^+^ and CD8^+^ T cells were pretreated with CD3/28 beads and then cultured for 72h in the presence of isolated WT or PD-L1 KO NETs. **(A, B)** Markers for T cell exhaustion were analyzed by flow cytometry were significantly upregulated on T cells in the presence of WT NET but not PD-L1 KO NETs. **(C, D)** T cell intracellular cytokines were analyzed by flow cytometry following fixation and permeabilization. T cells in the presence of WT NETs expressed significantly lower levels of intracellular cytokines compared to PD-L1 KO NETs. **(E, F)** Similarly, following culture with WT NETs, T cells were stained with metabolic markers, showing decreased mitochondrial activity, decreased glucose but increased lipid uptake in the presence of WT but not PD-L1 KO NETs. **(G, H)** T cells derived from PD-1 KO mice were treated in similar fashion and cocultured with WT NETs only and analyzed from Tim3 and Lag3 expression. These cells showed similar expression levels to WT T cells cultured with PD-L1 KO NETs. **(I, J)** Intracellular staining for TOX was performed showing decreased expression levels in T cells + PD-L1 KO NETs and PD-1 KO T cells + WT NETs. **(K, L)** Proliferation of CD4^+^ or CD8^+^ T cells in the presence of WT NETs or PAD4 supernatant. Bar graphs represent mean +/- SEM of at least 2 independent experiments, performed in duplicates or triplicates. *p < 0.05, **p < 0.01.

We have shown that NETs can directly promote T cell exhaustion *in vitro* (**Figure 4**) and that PD-L1 is embedded within NET chromatin. To further assess the role of PD-L1 in NETs on T cell exhaustion, we cultured normal T cells in the presence of isolated NETs from WT or PD-L1 KO mice *in vitro.* Following exposure of T cells to WT NETs, but not PD-L1 KO NETs, we found that markers of phenotypic T cell exhaustion were similar to those found *in vivo* within the NET-rich TME. T cells cocultured with WT NETs were found to upregulate surface markers of exhaustion including PD-1, Tim3 and Lag3, whereas T cells cultured with PD-L1 KO NETs had similar surface marker expression as control T cells (**Figures 6A, B**). In addition, co-cultures of T cells with WT NETs, but not PD-L1 KO NETs decreased T cell cytokine expression levels (**Figures 6C, D**). Furthermore, T cells cultured with WT NETs were found to change their metabolic profile leading to decreased mitochondrial function (TMRE), decreased mitochondrial mass (Mitotracker), decreased glucose (NDBG) and increased lipid (Bodipy) uptake (**Figures 6E, F**). T cells cultured with PD-L1 KO NETs had similar metabolic profile compared to control T cells. In addition, we showed that treatment of T cells from healthy donors with NETs from postoperative patients showed higher levels of phenotypic exhaustion compared to T cells treated with NETs from healthy individuals or no treatment (**Suppl. Figures 1B, C**).

We next tested the hypothesis that WT NETs cocultured with T cells derived from PD-1 KO mice would not promote exhaustion. PD-1 KO T cells failed to upregulate Tim3 and Lag3 in the presence of WT NETs with similar expression levels to T cells cocultured with PD-L1 KO NETs. (**Figures 5G, H**). Similarly, PD-1 KO T cells+WT NETs or T cells+PD-L1 KO NETs expressed lower levels of intracellular TOX, a protein known to be upregulated with T cell exhaustion, compared to T cells+WT NETs (**Figures 6I, J**). In addition, T cells+WT NETs had less proliferative capacity measured by CCK8 assay compared to T cells+PD-L1 KO NETs or PD-1 KO T cells + WT NETs (**Figures 6K, L**). These results are consistent with the hypothesis that exhaustion of T cells was due to the PD-L1 on NETs.

### Blocking of PD-L1 at Time of I/R Is Sufficient to Abrogate Tumor Growth

**Figure 7:**
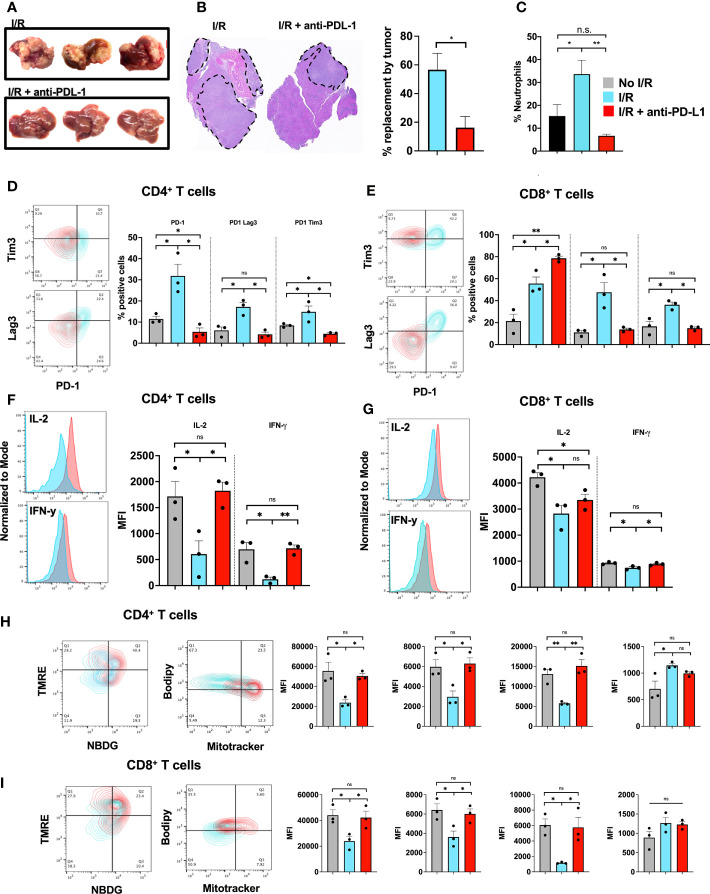
Blocking of PD-L1 at time of I/R is sufficient to abrogate tumor growth. **(A)** WT Mice underwent portal vein tumor infusion followed by I/R on day 6. Mice treated with I/R and anti-PD-L1 antibody had significantly decreased tumor burden at 3 weeks **(B)** H&E showed that animals that underwent IR and anti-PD-L1 treatment had smaller microscopic tumors. **(C)** Animals treated with anti-PD-L1 antibody had significantly decreased levels of neutrophils in the TME. **(D, E)** CD4^+^ and CD8^+^ T cells infiltrating tumors 3 weeks after liver I/R in WT mice expressed increase exhaustion markers **(F, G)** reduced T cell cytokine production **(H, I)** and altered metabolic function. Anti-PD-L1 antibody administration at time of I/R prevented these T cell changes. Bar graphs represent mean +/- SEM of two independent experiments, performed with three mice each. *p < 0.05, **p < 0.01.

The previous experiments have shown that T cells are more exhausted within a NET-rich TME, an effect mediated by PDL-1 expression on NETs. In addition, *in vitro* exposure of T cells to NETs, but not PD-L1 KO NETs, lead to similar exhaustive patterns comparable to a NET-rich TME. Following I/R, high levels of NETs and PD-L1 were found within the liver, this effect was reduced upon targeting of NETs with DNAse. These findings suggested that PD-L1 following I/R was mostly derived from NETs. We therefore treated cancer injected animals with two consecutive doses of anti-PD-L1 antibody immediately before and after I/R. We found that treatment with anti-PD-L1 reduced the size of the tumors as well as neutrophil infiltration at 3 weeks (**Figures 7A–C**). Similarly, treatment with anti-PD-L1 decreased the evidence of T cell exhaustion in the TME (**Figures 7D, E**). In addition, T cells in mice treated with anti-PD-L1 at time of I/R had significantly reduced levels of functional exhausted with increased cytokine (**Figures 7F, G**) and metabolic function (**Figures 7H, I**), respectively. These T cell functions in the TME resembled those from hepatic metastases in mice treated with DNAse (**Figure 2**).

## Discussion

Inflammation within the tumor microenvironment is considered one of the key mediators in tumor progression and metastasis and is often related to the infiltrating immune cells. Neutrophils have emerged as a key player in promoting tumor progression through release of NETs (13, 17, 18). However, there has been little direct evaluation of the crosstalk between NETs and other immune cells within the tumor microenvironment. In this current study we found that NETs have a direct effect on T cells by promoting phenotypic and functional exhaustion, which leads to decreased cytokine production, proliferative capacity and altered metabolism. This subsequently promotes the growth of tumor within the TME. We also found that the inhibitory ligand PD-L1 is embedded in NETs which leads to T cell dysfunction both *in vivo* and *in vitro* with reversal of the effect when DNAse or NETs from PD-L1 KO animals were used.

T cell exhaustion was originally identified during chronic infection in mice and was subsequently observed in humans with cancer. Exhausted T cells in the TME show overexpression of inhibitory receptors, decreased effector cytokine production, altered metabolic function and diminished anti-tumor immunity. The effect of T cell exhaustion leads to the failure of cancer elimination and tumor progression (19). One of the main mechanisms that promotes T cell dysfunction is the PD-L1-PD-1 axis. PD-1 was initially identified as a coinhibitory molecule on the surface of T lymphocytes. Interactions between PD-1 and its ligands, PD-L1 and PD-L2, activate the downstream signals of PD-1 and suppress T cell activation.

Under normal circumstances, T cells undergo metabolic reprogramming to glycolysis upon T cell receptor stimulation, which is required for T cell growth and effector function. Chronic activation of T cells promotes a metabolic switch for energy generation. With ongoing activation of the PD-1 signaling pathway, T cells express markers of phenotypic exhaustion. In addition, these cells are unable to engage in glycolysis, metabolism of branched-chain amino acids, however they increase the rate of fatty acid oxidation (FAO) (20, 21). In the current study we found that T cells in a NET-rich environment show decreased functioning mitochondria, decreased glucose but increased fatty acid uptake. These changes go along with the expression of markers of exhaustion and decreased cytokine expression. *In vitro*, upon treatment with NETs, CD4^+^ T cells show a similar metabolic phenotype compared to exhausted T cells in the TME. However, *in vitro* NET treated CD8^+^ T cells show enhanced mitochondrial function, increased glucose and lipid uptake but less mitochondrial mass. It is possible that CD8^+^ T cells are more resistant to chronic antigen stimulation and become hyperactive prior metabolic exhaustion. Interestingly, CD8^+^ T cells showed enhanced metabolic function upon treatment with NETs from PD-L1 ko animals. This suggests that both anti-CD3/anti-CD28 beads and the exposure to PD-L1 KO NETs can enhance the metabolic function above baseline.

The current study reveals a previously unidentified crosstalk between innate and adaptive immune cells, whereby NETs that are formed following surgical stress in the liver promote T cell exhaustion through PD-L1. Furthermore, NETs promoted an exhaustive state of T cells *in vitro* and targeting of PD-L1 in NET chromatin led to restoration of functional T cells.

Similarly, targeting of PD-L1 at time of surgery lead to decreased tumor burden and T cell exhaustion. We therefore believe that targeting NETs in the immediate perioperative period represents a potential strategy to restore functionally active T cells and promote anti-cancer immunity.

It is well known that the tumor microenvironment can actively suppress the function of invading T cells through increased expression of ligands for inhibitory receptors. It may have been assumed, therefore, that nonmalignant parenchymal tissues and innate cells, such as neutrophils, generally lacked the capacity to suppress infiltrating cells or, at the least, that such capacity was compromised. It is however well known that a high percentage of innate neutrophils and NETs found within solid tumors are associated with a poor prognosis (22). Neutrophils bear receptors for a number of neutrophil chemokines, including CXCL1 and CXCL2, that are formed following surgical stress but are also expressed on some tumor cells. These chemokines, in addition to a number of inflammatory mediators, can trigger neutrophil migration and NETosis. NETs themselves have several direct pro-tumorigenic effects including the ability to capture circulating tumors cells, promote their invasion and alter genes that favor metastatic growth (8, 12, 13, 17). A study by Teijeira et. al., has gone further and studied the interaction of NETs, cancer and adaptive immune cells. They showed that NETs may coat and shield tumor cells against cytotoxicity mediated by CD8^+^ T cells and NK lymphocytes. They hypothesize that this protective mechanism leads to armored tumor cells, with a loss of protection when DNAse is used. The effect of decreased tumor cytotoxicity in their study is likely mediated through direct contact inhibition, physical separation or obstruction by a negatively charged polymer (dsDNA), thereby sparing some of the malignant cells from immune attacks. It is also likely, that shielding of tumor cells from cytotoxicity is not the only mechanistic link between NETs and the hindrance of antitumor NK- and T cell-mediated immunity. Their findings also suggest that decreased lymphocyte migration to the TME upon exposure to NETs is one way of functional impairing these cells, even in the absence of tumor targets (14). These findings add to this current manuscript showing that NETs could have a dual role within the TME both by hindering T cells mediated tumor cytotoxicity through physical shielding as well as direct effects of NET chromatin by promoting T cell exhaustion.

NET chromatin itself harbors an extensive array of embedded proteins with varying stimulating or suppressive effects on adaptive and innate immune cells in human and mice (23). A large number of these proteins and potentially other neutrophil compounds that are adsorbed onto the NETs have preserved molecular functions. Proteases such as NE (neutrophil elastase), MPO, bactericidal cationic polypeptides, and other NET-adsorbed moieties have been described regarding their effect on cancer and immune cells. We have shown that NETs in the TME can directly foster tumor cell mitochondrial biogenesis and tumor proliferation induced *in vitro* in the presence of NETs was mediated by interaction of neutrophil elastase with tumor surface toll-like receptor (13).

A large spectrum of literature has proven the effect of neutrophils, neutrophil granules and NET components on T cell function. Studies performed on murine models have shown that T cell responses can be restored by depleting neutrophils, implying that neutrophils have a regulatory role (23–25). This suppression of T cell responses by neutrophils requires close contact and development of an immunological synapse (26). Furthermore, several forms of glomerulonephritis have shown that endogenous MPO, secreted by neutrophils, can suppress dendritic cells and T cell function. MPO inhibits ConA-induced proliferation of human T cells *in vitro* and T cells in MPO−/− mice have increased activation, proliferation and proinflammatory cytokine production (26). In addition, Sivanandham et al. has shown that CD4^+^ T cells in simian immunodeficiency virus infection become altered and undergo apoptosis once trapped within NET chromatin (27).

A wide array of literature supports the concept of accelerated tumor growth in the perioperative period. Several studies have linked surgical stress to activation of early and key components of the innate and adaptive immune system. In particular, platelet activation leading to a pro-coagulation state has been shown to accelerate tumor growth. In addition, surgical stress increases shedding of cancer cells into the circulation, promotes upregulation of endothelial adhesion molecules and also leads to changes within the cancer cell to increase migration and invasion. Furthermore, NETs have been found to trap circulating tumor cells and promote the growth of pre-existing micrometastasis (7, 28, 29). It is therefore important to not delay the initiation of systemic therapy in order to conquer the effects of surgery that can promote a pro-tumorigenic and immunosuppressive state.

Patients recovering from surgery have traditionally not received adjuvant chemotherapy due to the adverse effect of this intervention on wound healing and further immune suppression. However, the perioperative period potentially provides the opportunity to enhance the hosts immune system and attenuate the development of cancer recurrences (16).

A pilot study examining the effect of neoadjuvant administration of anti-PD-1 (nivolumab) in resectable non-small lung cancer patients has provided promising results. The authors observed major pathological responses in both PD-L1 positive and negative tumors. Patients receiving nivolumab showed increased proliferation of tumor infiltrating and peripheral T cells (30). The addition of anti-PD-L1 blockade to the perioperative chemotherapy treatment has since been evaluated in several clinical trials and found to be safe and effective (31, 32).

Such a protocol might well reduce the recurrence rate in patients that undergo resection of colorectal liver metastasis by targeting innate immune cells expressing PD-L1 that are found within the operative site after tumor resection.

In summary, this study gives new insights into the interaction of innate and adaptive immunity *via* PD-L1 contained within the NET chromatin capable of provoking exhaustion in nearby T cells. PD-L1 positive NETs can be readily observed *in vitro* and *in vivo* and are found in patients after surgical stress. This study is the first to implicate NET embedded PD-L1 as potential contributor to T cell exhaustion as an immune escape mechanism capable of augmenting metastatic tumor growth.

### Conclusion

The finding about interactions of NETs with tumor infiltrating T cells may have implications for understanding and designing therapy to target the immediate post-operative state of immunosuppression and tumor promoting effects of NETs. The current study has shown that targeting of PD-L1 following I/R decreases tumor burden and T cell exhaustion. This effect might a play an important role in humans undergoing surgery. Immediate post-operative treatment strategies targeting NETs together with anti-PD-L1 inhibition may be successful in decreasing tumor recurrence.

## Data Availability Statement

The raw data supporting the conclusions of this article will be made available by the authors, without undue reservation.

## Ethics Statement

The studies involving human participants were reviewed and approved by University of Pittsburgh: STUDY19010199. The patients/participants provided their written informed consent to participate in this study. The animal study was reviewed and approved by University of Pittsburgh: Protocol #: 20036884.

## Author Contributions

CK, HY, KM, and ST contributed to conception and design of the study. CK, HY, and KM performed the experiments. CK and HY performed the statistical analysis. CK, RS, and ST wrote the first draft of the manuscript. CK, HY, KM, DG, and ST wrote sections of the manuscript. DG, RS, and ST performed the final revision of the manuscript. All authors contributed to manuscript revision, read, and approved the submitted version.

## Funding

The University of Pittsburgh holds a Physician-Scientist Institutional Award from the Burroughs Wellcome Fund, ST and CK. This work was supported by a Community Liver Alliance (CLA) Grant (ST). ST was supported in part by NIH/NIDDK Digestive Disease Research Core Center grant P30DK120531, Univerity of Pittsburgh Competitive Medical Research Fund (CMRF) Grant 2020, and National Institute of Health (NIH) 1S10OD019973-01 (Center of Biological Imaging – Nikon A1).

## Conflict of Interest

The authors declare that the research was conducted in the absence of any commercial or financial relationships that could be construed as a potential conflict of interest.

## Publisher’s Note

All claims expressed in this article are solely those of the authors and do not necessarily represent those of their affiliated organizations, or those of the publisher, the editors and the reviewers. Any product that may be evaluated in this article, or claim that may be made by its manufacturer, is not guaranteed or endorsed by the publisher.

## Supplementary Material

Supplementary Figure 1
**(A)** Neutrophils from healthy or postoperative patients were treated with PMA and stained for cit-H3 and PD-L1, respectively. Co-localization of cit-H3 and PD-L1 are indicated by white arrows. **(B, C)** CD4^+^ or CD8^+^ T cells were isolated from healthy individuals, cocultured with NETs from healthy volunteer donors or postoperative patients and stained for markers of exhaustion. **(D)** Exhaustion surface marker expression levels were determined on naïve splenic CD4^+^ T cells after culture with WT NETs or WT NETs + DNAse using flow cytometry. (n=3). Bar graphs represent mean +/- SEM of two independent experiments (A/C), *p < 0.05, **p < 0.01.
